# Current Understanding of BRAF Alterations in Diagnosis, Prognosis, and Therapeutic Targeting in Pediatric Low-Grade Gliomas

**DOI:** 10.3389/fonc.2015.00054

**Published:** 2015-03-03

**Authors:** Catherine Louise Penman, Claire Faulkner, Stephen P. Lowis, Kathreena M. Kurian

**Affiliations:** ^1^Brain Tumour Research Group, Institute of Clinical Neurosciences, University of Bristol, Bristol, UK; ^2^Bristol Genetics Laboratory, Pathology Sciences Southmead Hospital, Westbury on Trym, Bristol, UK; ^3^Department of Paediatric Oncology, Bristol Royal Hospital for Children, Upper Maudlin Street, Bristol, UK

**Keywords:** BRAF, glioma, brain tumor, diagnostic biomarker, prognostic biomarker, therapeutic targeting

## Abstract

The mitogen-activated protein kinase (MAPK) pathway is known to play a key role in the initiation and maintenance of many tumors as well as normal development. This often occurs through mutation of the genes encoding RAS and RAF proteins which are involved in signal transduction in this pathway. BRAF is one of three RAF kinases which act as downstream effectors of growth factor signaling leading to cell cycle progression, proliferation, and survival. Initially reported as a point mutation (*V600E*) in the majority of metastatic melanomas, other alterations in the *BRAF* gene have now been reported in a variety of human cancers including papillary thyroid cancer, colon carcinomas, hairy cell leukemia, and more recently in gliomas. The identification of oncogenic mutations in the *BRAF* gene have led to a revolution in the treatment of metastatic melanoma using targeted molecular therapies that affect the MAPK pathway either directly through BRAF inhibition or downstream through inhibition of MEK. This review describes the molecular biology of BRAF in the context of pediatric low-grade gliomas, the role of BRAF as a diagnostic marker, the prognostic implications of BRAF, and evidence for therapeutic targeting of BRAF.

## Introduction

**Figure 1:**
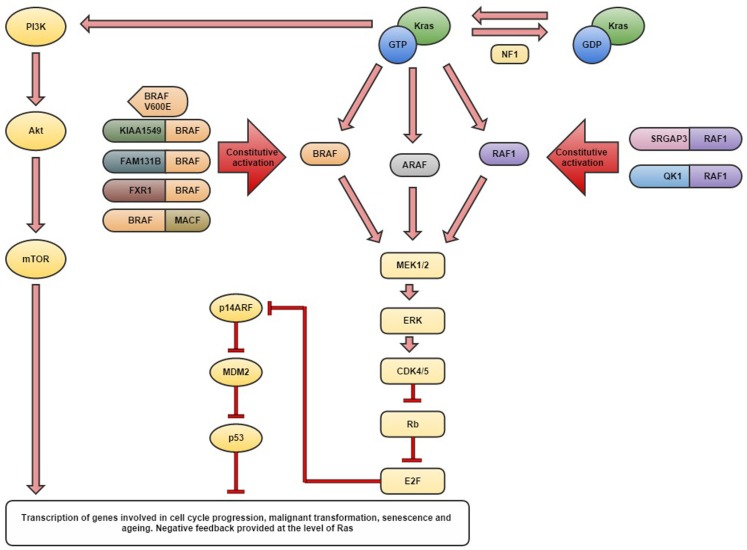
**Schematic diagram detailing the currently identified aberrations in the MAPK pathway genes thought to be responsible for the development of pilocytic astrocytomas**.

Pediatric low-grade gliomas (LGGs) represent the most common central nervous system (CNS) tumors of childhood, with pilocytic astrocytomas (PAs) being the most prevalent, accounting for 17% of brain and spinal neoplasms in children age 0–14 years (1, 2) (see Table 1). For the purposes of this review, the term LGGs is used to describe a heterogeneous group of tumors including both WHO grade I and II neoplasms (see Table 1) (3). The incidence varies from 0.26 to 1.79/100,000 depending on the histology and geographical region, with an overall higher incidence in males (1). Survival is variable with a 5-year overall survival in PAs being reported as high as 100%, compared to 45% in diffuse fibrillary astrocytoma (DA) (1, 4). PAs are relatively benign slow growing tumors occurring most commonly within the cerebellum, but may also arise along the optic tract, in the hypothalamus and brain stem where they are difficult to fully resect (5). Complete resection has been reported in 94% of cerebellar PAs compared with only 3.2% of hypothalamic and chiasmatic tumors, with an overall tumor recurrence rate of 19% for PAs (6, 7). PAs of the optic tract are common in the hereditary syndrome neurofibromatosis type 1 and are commonly associated with defect in the *NF1* gene (8). *NF1* loss may activate the mitogen-activated protein kinase (MAPK) pathway through RAS, which in turn may activate BRAF and the PI3K pathway (Figure 1) (8–10). DAs have been reported to represent approximately 3% of pediatric gliomas, although they more commonly occur in adults (11). Within the pediatric population, the higher grade gliomas, anaplastic astrocytoma (AA), and glioblastoma multiforme (GBM) account for approximately 5 and 6.5% of pediatric gliomas, respectively (11).

**Table 1:** **Histological subgroups of low-grade and high-grade gliomas demonstrating in which tumors the *BRAF* gene fusion has been identified**.

Histological subgroup	WHO grade	*KIAA1549:BRAF* fusion described	Average% fusion positive	Other *RAF* fusion described	*BRAF V600E* described	Average% *BRAF V600E* positive	Reference
Pilocytic astrocytoma	I	Yes	77.2	*FAM131B:BRAF*, *SRGAP3:RAF1*, *QK1:RAF1*	Yes	6.2	(9, 12–19)
Pilomyxoid astrocytoma	II	Yes	62.5		Yes	5.0	(9, 13, 14, 19, 20)
Diffuse fibrillary astrocytoma	II	Yes	3.0	*FAM131B:BRAF*	Yes	8.1	(13, 14, 17–19)
Anaplastic astrocytoma	III	No	0		Yes	15.9	(15, 17, 18, 21)
Glioblastoma multiforme	IV	No	0		Yes	9.4	(15, 17, 18, 21)
Pleomorphic xanthoastrocytoma	II	Yes	55.6		Yes	50.8	(13, 14, 17–19)
Ganglioglioma	I/II	Yes	25.3	*BRAF:MACF*, *FXR1:BRAF*	Yes	20.7	(9, 14, 17–19)
Dysembryoplastic neuroepithelial tumor	I	No	0		No	0	(9, 14, 18)
Desmoplastic infantile astrocytoma/glioma	II	No	0	*FXR1:BRAF*	Yes	8.5	(18, 22)

### Molecular biology of BRAF

#### BRAF fusion

Until recently, the molecular biology of pediatric LGGs was relatively unknown (see Figure 1 and Table 2). The genetic aberrations commonly seen in adult gliomas including *p53*, *PTEN*, *CDK4*, and *p16* have not been identified in low-grade pediatric gliomas suggesting that they may be genetically distinct (23).

**Table 2:** **Gene fusions involving members of the MAPK pathway including *BRAF* and in which low-grade gliomas the fusions have been identified**.

MAPK pathway gene	Gene fusion	Exon variant	Tumors fusion is present in	WHO grade	Reference
BRAF	*KIAA1549:BRAF*	exons 16:9, exons 15:9, exons 16:11	PA, PMA, PXA, GG, SGCA, DA (A and ODG)	I, II	(9, 13, 15, 24, 32, 34)
BRAF	*KIAA1549:BRAF*	exons 15:8	PA	I	(12)
BRAF	*KIAA1549:BRAF*	exons 16:10	PA	I	(26)
BRAF	*KIAA1549:BRAF*	exons 15:11, exons 17:10	PA, LGG	I, II	(27)
BRAF	*KIAA1549:BRAF*	exons 18:10	PA	I	(9, 13)
BRAF	*KIAA1549:BRAF*	exons 19:9	PA	I	(13)
BRAF	*FAM131B:BRAF*	exons 2:9, exons 3:9, exons 2:10	PA	I	(12)
BRAF	*FXR1:BRAF*	Not reported	DA	II	(19)
BRAF	*BRAF:MACF*	Not reported	GG	II	(19)
RAF1	*SRGAP3:RAF1*	exons 12:10	PA	I	(16)
RAF1	*SRGAP3:RAF1*	exons 12:7, exons 11:9	PA	I	(12)
RAF1	*SRGAP3:RAF1*	exons 11:8	PA	I	(13)
RAF1	*QK1:RAF1*	Not reported	PA	I	(19)

*PA, pilocytic astrocytoma; PMA, pilomyxoid astrocytoma; PXA, pleomorphic astrocytoma; DA, diffuse fibrillary astrocytoma; A, grade II astrocytoma not further specified; GG, ganglioglioma; SGCA, subependymal giant cell astrocytoma; ODG, oligodendroglioma; LGG, low-grade gliomas not further specified. A and ODG were found to have the *KIAA1549:BRAF* fusion in 6 of 118 cases and were thought to be misdiagnosed PA as they all followed a particularly benign clinical course (15)*.

In 2008, several papers identified a genetic defect in the *BRAF* gene thought to be responsible for constitutive activation of the MAPK pathway and thus development of PAs in children (15, 24, 25). Jones at al. described a tandem duplication at 7q34 resulting in fusion of the previously uncharacterized gene *KIAA1549* and the *BRAF* gene to create a novel fusion oncogene in approximately 66% of PAs (15). Other less common fusion variants included exon 15:exon 9, exon 16:exon 11, exon 16:exon 10, exon 15:exon11, exon 17:exon 10, and exon 18:exon 10 (see Table 2) (9, 15, 26, 27). All fusions were found to have constitutive BRAF kinase activity and transforming ability in NIH3T3 cell lines (15). The constitutive kinase activity of KIAA1549:BRAF fusion oncoprotein is due to the loss of the BRAF N-terminal auto-inhibitory domain which usually regulates BRAF activity (15, 28). Lin et al. demonstrated that the KIAA1549:BRAF fusion is transcribed from the *KIAA1549* gene promoter and thus is expressed at higher levels than wild-type BRAF, so the fusion oncoprotein is not only constitutively active but also over expressed giving two mechanisms of aberrant activity (27). Investigation into the effects of the oncogenic fusion protein formed by KIAA1549 and BRAF has revealed that the constitutive activation regulates neuroglial cell growth in a cell-type-specific manner causing proliferation of neural stem cells but not mature astrocytes (29). In certain studies, the *KIAA1549:BRAF* fusion gene was also found to function through MEK-dependent activation of both MAPK and mTOR pathways and the injection of neural stem cells containing the fusion were sufficient to induce glioma-like lesions in mice (29). Whereas other studies report that expression of the KIAA1549:BRAF fusion protein alone is not sufficient for gliomagenesis and instead results in senescence (29–31). More recently, several novel gene fusions have been discovered which may account for MAPK activation in PAs which do not harbor the *KIAA1549:BRAF* fusion variants, summarized in Table 2 (12, 13, 16, 26). A further novel mutation at codon 209 in the *GNAQ* gene has been described in a single case of PA (32). *GNAQ* encodes a Gα subunit of the guanine nucleotide-binding protein receptor involved in signaling upstream of RAS (33). Mutations in the *GNAQ* gene are thought to lead to constitutive activation of the MAPK pathway, independent of BRAF (33).

#### BRAF V600E mutation

Characterization of mutant proteins has revealed a mutation hotspot resulting in a valine to glutamate substitution at position 600, often referred to as *BRAF V600E* in a range of tumor types (20, 35–39). The BRAF protein and its close relation RAF1 (CRAF) are usually subject to auto-regulation through different N-terminal auto-inhibitory domains (28). The oncogenic *V600E* mutation lies within the activation segment disrupting the auto-inhibitory mechanism and converting BRAF into its active form thus allowing constitutive activation of the MAPK pathway (40). Gronych et al. demonstrated that overexpression of the mutant BRAF V600E kinase domain alone induced tumor formation with clinical and histological features of PAs whereas the full length V600E mutant protein which still contained the autoregulatory domain did not give rise to tumors (31). In inkk4a/ARF-deficient mice, the full length BRAF V600E could induce tumorigenesis but these more closely resembled high-grade astrocytomas (31). Lyustikman et al. demonstrated a causal relationship between constitutive activation of RAF1, and thus the downstream MAPK signaling pathway, and glioma formation in mice (30). Activation of RAF1 alone induced hyperplastic lesions in Ntv-a mice, yet such lesions did not progress without concomitant loss of ARF (see Figure 1). With loss of ARF and the *RAF1* mutation, mice developed lesions similar to glioblastoma, yet without ARF loss, small hyperplastic lesions developed which may represent tumors more similar to PAs (30). Huillard et al. reported that the presence of *BRAF V600E* alone was insufficient for gliomagenesis and a concomitant homozygous deletion of *CDKN2A* (which encodes P14ARF and P16INK4A) was required for the development of astrocytomas from neural progenitor cells (Figure 1) (41). It was demonstrated that expression of BRAFV600E may cause transformation when combined with loss of *CDKN2A* in human neural progenitor cells and the resultant tumors displayed the histology of malignant astrocytomas (41).

Outwith the glioma biology field, it has been proposed that the *BRAF V600E* mutation may be responsible for the induction of growth arrest and senescence in melanocytic naevi, in a process termed “oncogene-induced senescence” whereby melanocytic naevi can remain in growth arrest for a lifetime (42). Oncogene-induced senescence has been described when *BRAF V600E* mutation was introduced with a lentiviral vector into human neurospheres derived from the cerebral cortex of first trimester human fetal autopsy specimens (43). Neurosphere cells initially underwent transformation, subsequently followed by senescence with expression of senescence-associated markers (44). Interestingly, Jacob et al. have described that the majority of PAs are senescent and that this effect is triggered through the p16ink4a pathway following aberrant MAPK activity (45). Moreover, Hawkins et al. overexpressed BRAF in hTERT immortalized human astrocytes and found that this caused growth arrest and senescence with associated DNA damage (32).

### BRAF as a diagnostic marker

#### BRAF fusion

The *KIAA1549:BRAF* fusion has been reported in a range of PAs (59–90%) and so it is increasingly used as a diagnostic marker for PAs, where neuropathological distinction from malignant glioma can be difficult (46, 47). The literature is divided on the incidence of *BRAF* fusions in other pediatric LGGs which has implications for testing of these entities (9, 14, 22, 43, 46, 47). A few studies have shown a lack of *BRAF* fusions in ganglioglioma (GG), desmoplastic infantile GG/astrocytoma, dysembryoplastic neuroepithelial tumor, pilomyxoid astrocytoma (PMA), and pleomorphic xanthoastrocytoma (PXA) (9, 22, 43, 46, 47). By contrast, Horbinski et al. describe *BRAF* rearrangements in up to 15% of non-pilocytic LGGs including GG, PMA, and PXA, but not DAs (14). In a larger study by Hawkins et al. of pediatric low-grade astrocytomas including 105 PAs, 6 PMAs, 71 diffuse astrocytomas, and 4 unspecified low-grade astrocytomas, *BRAF* fusions were described in 62% of PAs, 67% of PMAs, 37% of diffuse astrocytomas, and 50% of unspecified low-grade astrocytomas.

Cin et al. screened 125 primary PAs for the known *KIAA1549:BRAF* fusion, the *SRGAP3:RAF1* fusion, and also described a novel fusion between the otherwise uncharacterized gene product FAM131B and BRAF (12). In this study, fusions were identified in 82% of cerebellar PAs and 57% of non-cerebellar PAs, and a further 4.8% of tumors contained the *BRAF V600E* mutation (12). Cykowski et al. report the use of the *KIAA1549:BRAF* fusion and *TP53* to distinguish PAs with atypical features from the highly malignant glioblastoma (48). Korshunov et al. describe the use of the *KIAA1549:BRAF* fusion to distinguish PAs from *R132H IDH1* mutation positive diffuse astrocytoma counterparts with high specificity, with a consistent lack of *R132H IDH1* mutation positivity in PAs (49).

#### BRAF V600E mutation

In a large cohort study of CNS tumors ranging from grade I to grade IV cancers, *BRAF V600E* mutation occurred most frequently in 66.7% of PXAs, but was also found at lower levels in PAs, GGs, and malignant gliomas (17). In another cohort study of over 1,300 CNS tumors, 66.7% of PXAs, 18% of GGs, and 9% of extra-cerebellar PAs harbored the *BRAF V600E* mutation (4). Further mutations have been found in the *BRAF* gene in gliomas including a 3bp insertion at codon 598 which mimics the *V600E* mutation (16). Further findings indicate that aberrations of *MYB* and *MYBL1* may help distinguish LGGs from PAs as these aberrations were found in 68% of diffuse astrocytomas but 0% pilocytic tumors (42). The *BRAF V600E* mutant and the *KIAA1549:BRAF* fusion are generally mutually exclusive with only a few cases reported with both the fusion and the *V600E* mutation (12, 14, 32).

In summary, although currently assessment of the *BRAF* fusion is of most diagnostic use in posterior fossa PAs, and the *BRAF V600E* mutation is more prevalent in PXAs, both of these alterations have been described to varying degrees in other pediatric LGGs including DA.

### Prognostic implications of BRAF status

Although there is much debate over the association of the fusion status with outcome, it remains generally accepted that the patient age, location of the tumor, and extent of resection are the most important prognostic indicators (6, 7, 14, 50). However, the presence of the *KIAA1549:BRAF* fusion positive compared to fusion negative pediatric LGGs has been associated with improved outcome in two studies (14, 32) and reported to have no effect on outcome in four (6, 15, 27, 34). For example, in one of the positive studies, the *KIAA1549:BRAF* fusion was associated with better clinical outcome in the large cohort by Hawkins et al. of pediatric low-grade astrocytomas including PAs, PMAs, and diffuse astrocytomas (32). In this study, all LGGs included were extra-cerebellar, incompletely resected (less than 75% resection), and followed up for more than 1 year (32). The overall findings were that the 5-year progression-free survival (PFS) was 61% in *BRAF* fusion positive tumors compared to 18% in fusion negative tumors (with PFS defined as greater than 25% increase in tumor volume on consecutive MRI scans) (32). Moreover, multivariate analysis revealed that *BRAF* fusion was an independent prognostic factor in incompletely resected LGGs (32). Significantly, all fusion negative PA patients under the age of 18 months experienced progression of their tumors within 8 years of diagnosis, and three of five patients in this group died within the study period of 16 years (32). In the second positive study, Horbinski et al. also report the presence of *BRAF* rearrangements as a positive prognostic marker in a cohort of LGGs including PAs, GGs, PMAs, PXAs, diffuse astrocytomas, oligodendrogliomas (ODGs), subependymal giant cell astrocytomas (SGCAs), dysembryoplastic neuroepithelial tumors, and LGGs not otherwise specified (14). *BRAF* rearrangement was found to be associated with longer PFS and decreased risk of death, only one fusion positive patient died within the 20-year follow-up period (14).

However, it is possible that the *BRAF* fusion is more a diagnostic marker of the PA which has an inherent better prognosis, as other studies of PA demonstrate no survival advantage in *BRAF* fusion positive cases compared with negative cases (6, 15, 27, 51). In a small cohort of PAs, the presence of the *BRAF V600E* mutation has been reported to be significantly associated with both diffusely infiltrating architecture and increased risk of progression in LGGs (14, 52).

The association with location is less controversial with mounting evidence that the *BRAF V600E* mutation is more common in supratentorial PAs and the *KIAA1549:BRAF* fusion being more common in posterior fossa PAs (14, 33, 34, 53, 54). However, further evidence from Hawkins et al. looking specifically at supratentorial LGGs found that midline tumors which are usually unresectable were more likely to harbor the *KIAA1549:BRAF* fusion (65% cases) compared to only 11% of lobar tumors which were found to be *BRAF* fusion positive (32). In this study, it was also found that *BRAF* fusion positive tumors had better 5-year PFS irrespective of tumor histotype or location with 5-year PFS of 65% in fusion positive PAs compared to 17% for fusion negative tumors (32).

### Therapeutic targeting of BRAF and MAPK pathway

#### BRAF and MAPK inhibitors in cell lines and animal models

##### Non-central nervous system cell lines and xenografts

Therapeutic manipulation of the BRAF and MAPK pathway has been extensively investigated in many tumor types (see Figure 1 and Table 3). For example, sorafenib is a potent RAF1 inhibitor with action against BRAF, PDGFRβ, and VEGFR-3 (55). Sorafenib has been shown to act against both wild-type and *BRAF V600E* mutant cell lines and colon, breast, and non-small cell lung cancer xenograft models (55, 56). Inhibition of tumor growth in these models was not associated with any apparent toxicities and appeared to be elicited through abrogation of MAPK signaling; however, the drug did not discriminate between those cells with aberrant MAPK activity and wild-type cells (55). Tsai et al. report the discovery of a selective inhibitor PLX4720 of *BRAF V600E* whose cytotoxic effects are specific to mutant cells only (57). This PLX4720 drug induced cell cycle arrest and apoptosis in *V600E* mutant cell lines derived from colon carcinoma, and melanoma cells and yet had minimal effects in other cancer cell lines which did not possess the *V600E* mutant, including RAS-mutant colon carcinoma cells, large cell lung cancer cells, and metastatic melanoma (57). Moreover, a dose of 20 mg/kg PLX4720 was given orally for 14 days to mice with colorectal carcinoma xenografts which harbored the *V600E* mutation, achieving tumor regression below palpable levels in four of nine mice (57). Solit et al. demonstrated that *BRAF* mutant cells, unlike their wild-type relatives, are dependent on MEK signaling for growth and survival and therefore *BRAF V600E* mutant cells had enhanced sensitivity to MEK inhibitors compared to wild-type *BRAF* cells and *NRAS* mutant cells (58). Furthermore, daily treatment with the MEK inhibitor PD0325901 in *BRAF V600E* mice xenografts showed complete suppression of growth, whereas wild-type xenografts were insensitive to MEK inhibition (58).

**Table 3:** **Small molecule inhibitors currently in clinical trials to evaluate safety and efficacy in pediatric low-grade gliomas and other tumors**.

Drug	Target	Tumor type	Phase	Problems reported	Reference/trial identifier
Dabrafenib^a^	BRAF	*BRAF V600E* positive tumors including HGG and LGG	I/IIa	Rash, palmar–plantar syndrome, skin thickening, headaches, GI disturbances, arthralgia, alopecia, fever, lethargy, squamous cell carcinomas, photosensitivity, kidney dysfunction, pancreatitis, and loss of fertility	NCRN511 (67, 68)
Dabrafenib^a^	BRAF	*BRAF V600E* positive solid tumors	II	As above	NCT01677741
Trametinib (in combination with dabrafenib)	MEK 1/2	*BRAF V600E* positive solid tumors	II	No study results	NCT02034110 (69)
Selumetinib (AZD6244)^a^	MEK 1/2	LGG	I/II	No study results	NCT01386450
Selumetinib (AZD6244)^a^	MEK 1/2	LGG	I/II	No study results	NCT01089101
Everolimus (RAD001)^a^	mTOR	*NF1*-recurrent or refractory gliomas	II	Myelosuppression, stomatitis, rash, fatigue, nausea, headaches, pneumonitis, amenorrhea, loss of fertility, fluid retention, and blood sugar disturbances – requires monitoring (from CRUK website, http://www.cancerresearchuk.org/about-cancer/cancers-in-general/treatment/cancer-drugs/everolimus)	NCT01158651 (70)
Everolimus (RAD001)^a^	mTOR	Recurrent or refractory LGG	II	No study results	NCT01734512
Everolimus (RAD001)^a^	mTOR	Recurrent or refractory LGG	II	No results	NCT00782626
Sorafenib (BAY 43-9006)^a^	RAF kinases (RAF1 > BRAF), VEGF, and PDGFR	Recurrent or progressive low-grade astrocytomas	II	Raised alanine aminotransferase, raised aspartate aminotransferase, diarrhea, mucositis, headache, rash, dry skin, hand–foot–skin syndrome, fatigue, alopecia, anorexia, hypophosphatemia, and lymphopenia	NCT01338857 (71–73)
	RAF kinases (RAF1 > BRAF), VEGF, and PDGFR	Metastatic melanoma	II	Fatigue, pain, gastrointestinal disturbance (diarrhea), and dermatological reactions (palmar–plantar syndrome, rash), increased risk of bleeding, and loss of fertility	(66)
Vemurafenib (PLX4032/PLX4720)^a^	BRAF	Recurrent or refractory *BRAF V600E* mutant gliomas	0	No results	NCT01748149 (74)
Vemurafenib (PLX4032/PLX4720)^a^	BRAF	Metastatic melanoma	II/III	Arthralgia, rash, fatigue, alopecia, keratoacanthoma, squamous cell carcinoma, photosensitivity, nausea, and diarrhea	(75–77)
Vemurafenib/RO5185426 (BRIM-P trial)^a^	BRAF	Stage IIIc/IV melanoma with *BRAF V600E* mutation	I	No results	NCRN324

*^a^Drugs marked are known to cross the blood–brain barrier and thus may be of use in gliomas. Information regarding trials can be found at http://clinicaltrials.gov/ using the trial identifiers quoted. Accessed: 27/08/2014 (78)*.

##### Central nervous system cell lines and xenografts

In gliomas, Huillard et al. report significantly increased survival in mice transplanted with human *V600E* mutated astrocytoma cells when treated with the first generation BRAF inhibitor vemurafenib (PLX4720) (41). In these intracranial astrocytoma models, the overall tumor size was decreased with BRAF inhibitor treatment, whereas no response was observed when administered with the wild-type BRAF control treatment (41). Moreover, Nicolaides et al. demonstrated efficacy of the BRAFV600E inhibitor PLX4720 in reducing tumor growth and increasing survival in the *BRAFV600E* mouse model, whilst being ineffective in the wild-type xenograft models (21). A MEK inhibitor AZD6244 was tested against the pediatric pre-clinical testing *in vitro* panel with the highest *in vitro* concentrations inhibiting growth by 50% in 5 of 23 cell lines derived from a variety of pediatric cancers including glioblastoma, rhabdomyosarcoma, rhabdoid tumors, Ewing’s sarcoma, neuroblastoma, acute lymphoblastic leukemia, acute myeloid leukemia, anaplastic large cell lymphoma, and non-Hodgkin’s lymphoma (59). When the MEK inhibitor AZD6244 was tested against PA xenograft models, complete regression was observed in the *BRAF V600E* mutant xenograft whereas tumor progression was observed in wild-type *BRAF* xenografts (59).

See et al. further report the use of MEK inhibitors (AZD6244 or PD0325901) in *NF1*-deficient GBM cell lines with resultant growth inhibition in a subset of cells (both agents) and *in vivo* in nude mouse xenograft models (PD03125901) (8). This may indicate that other tumors driven by *NF1* loss, including PA associated with neurofibromatosis, may be amenable to small molecule inhibition of downstream targets in the MAPK pathway (8).

Paradoxical activation, whereby the BRAF inhibitor activates the MAPK pathway, has been described in cells with wild-type *BRAF* treated with a first generation BRAF inhibitor and appears to be caused by transactivation of RAF dimers to initiate ERK signaling (60). Moreover, Hatzivassilliou et al. have called for careful patient selection in RAF inhibitor trials due to the finding of paradoxical activation of the MAPK pathway in *V600E* negative cells through RAF1 priming (61). Second generation BRAF inhibitors, such as PLX PB3, on the other hand, have been found to have equal action against both the mutant and fusion protein (62). These BRAF inhibitors do not cause paradoxical activation in cells containing wild-type *BRAF* resulting in the desired effects of MAPK pathway inhibition and decreased proliferation (62).

#### BRAF and MAPK inhibitors in clinical cohorts

Currently, there are few trials involving MAPK inhibiting agents and pediatric LGGs (ongoing trials are summarized in Table 3).

##### BRAF and MAPK inhibitors in melanoma

Much of the clinical research into agents such as BRAF or MEK inhibitors has been performed on *V600E* positive metastatic melanomas in adults (63–66). Although these tumors are distinct from pediatric gliomas, information gained on safety, toxicity, dosage, and efficacy of these novel agents can help us understand the potential of these new drugs in treating these LGGs arising with similar molecular alterations (39, 43).

Bollag et al. report 26/36 metastatic melanoma patients achieving a partial or complete response to maximum tolerated dose of vemurafenib in a phase I clinical trial (78). The main problem reported in this trial was the development of multiple skin squamous cell carcinomas in 31% patients, a finding which has been replicated in several other BRAF inhibitor trials (64, 75, 78). A further phase II study in patients with metastatic melanoma reported an overall response rate of 53% with vemurafenib, with a median overall survival 15.9 months compared to 6–10 months with standard therapy (77). A phase III clinical trial of vemurafenib compared to the standard therapy dacarbazine in previously untreated metastatic melanoma with known *BRAF V600E* mutation demonstrated an increase in overall survival of 20%, and a reduction of 63% in the risk of death in the vemurafenib group (75). Common adverse effects reported with the BRAF inhibitor were again cutaneous squamous cell carcinoma, as well as those detailed in Table 3. The overwhelming success of treatment of metastatic melanoma with this novel agent led to the decision that patients originally randomized to dacarbazine should be allowed to cross over into the other treatment group (75).

##### BRAF and MAPK inhibitors in pediatric glioma

In pediatric low-grade astrocytomas, a recent phase II trial of the multikinase inhibitor sorafenib (Table 3) was discontinued early due to the unexpected acceleration of tumor growth (73). Eleven patients with progressive LGG following at least one chemotherapy treatment were recruited, three of which were positive for *NF1* and five who possessed the *BRAF* gene alteration *KIAA1549:BRAF* (73). The pathology included pilocytic and PMAs, DA, GG, and LGG not otherwise specified (73). The median time to tumor progression was 2.8 months in nine patients, and after three cycles of treatment, all but two participants had experiences disease progression (73). Sorafenib was well tolerated but the striking finding was the rapid progression of these usually slow growing tumors leading to the discontinuation of this trial (73).

#### Resistance to inhibitors

##### Resistance to inhibitors in melanoma

Duration of response to BRAF inhibitors in metastatic melanoma has been recorded as ranging from 2 to 18 months (64). Several diverse mechanisms for this resistance have been proposed, including receptor tyrosine kinase and NRAS upregulation (79), dimerization of wild-type or fused BRAF (60, 62, 80), and through the other RAF kinases most notably RAF1 (60, 61, 81–83). *RAF1* mutations lead to increased homodimerization and heterodimerization with BRAF following exposure to the RAF inhibitor PLX4032 (82). Increased dimerization leads to the development of resistance to PLX4032, known clinically as vemurafenib, in A725 cells (82). Emery et al. report novel *MEK 1* mutations arising as a consequence of treatment of metastatic melanoma with the MEK inhibitor AZD6244 (84). *MEK* mutations were found either in the allosteric drug binding site or in one of the function domains and were shown to cause pharmacological resistance to both AZD6244 and cross resistance to the BRAF inhibitor PLX4720 (84). Other *MEK1* mutations have been described as a mechanism conferring resistance in metastatic melanoma from a patient with an initial near-complete response to PLX4032 with subsequent relapse in week 16 of treatment leading to rapid disease progression and death (85). Further mechanisms of developing resistance have been described including increased COT expression (a gene transcribed from MAP3K8 which acts as a MAPK agonist) which increases phosphorylation of ERK and MEK in a RAF-independent fashion leading to PLX4720 resistance in metastatic melanoma cell lines (86). Furthermore, this resistance extended to MEK inhibition with AZD6244 indicating that COT may act through both MEK-dependent and -independent mechanisms (86).

#### Overcoming limitations through combination therapy

Combined BRAF and MEK inhibition has been attempted as a method of overcoming resistance (83). Combined therapy using both a BRAF inhibitor (PLX4720) and a MEK inhibitor (AZD6244) has demonstrated the ability to overcome resistance to MEK inhibition in metastatic melanoma cell lines (78, 80). Fragomeni et al. demonstrated the ability to induce complete regression of *V600E* positive xenograft metastatic melanomas through combining CRM1 and BRAF inhibition (87). CRM1 is known to play a role in melanoma proliferation, but has not been found to be part of tumor development or progression in LGGs to date (87). Flexible switching between RAF isoforms as a mechanism of resistance to RAF inhibitors may be overcome through co-targeting of MEK and IGF1R or PI3K in BRAF inhibitor-resistant melanoma cells (BRAF inhibitor-SB-590885) (25). A single RAF isoform was found to be sufficient to activate downstream signaling and knock down of two isoforms was not sufficient to induce cell cycle arrest seen when all three isoforms were blocked (25).

Huillard et al. demonstrated the increased efficacy of dual therapy combining the BRAF V600E inhibitor PLX4720 and the CDK4/6 inhibitor PD0332991 in human GBM xenograft models compared to either agent as a monotherapy (41). Combination of these two agents directly targeted two distinct enzymatic activities and was shown to suppress the paradoxical stimulation of Akt (Figure 1) which occurs with treatment of xenografts with PLX4720 alone (41).

In clinical cohorts, the timing and order of combination therapy has been found to impact on outcome, with prolonged survival being seen in patients receiving MEK inhibitors before BRAF inhibitors compared to the reverse sequence of drug administration (88). Moreover, there was increased tolerability of combination therapies in this sequence, with a lower incidence of the development of neoplastic cutaneous skin lesions (88). The testing of agents in combination to overcome resistance is interesting in the pre-clinical setting. However, at present, they cannot be considered for clinical trials in pediatric patients due to regulatory issues.

## Conclusion

Currently, assessment of the *KIAA1549 BRAF* fusion is most useful as a diagnostic biomarker in posterior fossa PAs. Although the *BRAF V600E* mutation is more prevalent in PXAs, it has been described to varying degrees in other pediatric LGGs and is of less diagnostic use. In terms of prognosis, the *KIAA1549 BRAF* fusion has been described as an independent positive prognostic biomarker in low-grade pediatric gliomas, irrespective of tumor type. As more targeted molecular therapies become available, there will be increased pressure for testing for both the *BRAF V600E* and the *KIAA1549 BRAF* fusion in order to predict treatment options in pediatric LGGs. However, it will be important to learn from lessons from therapeutic targeting of BRAF in metastatic melanoma, whereby targeting one pathway may induce resistance through upregulation of other pathways. Initial cell line work suggests that a combinatorial approach may be more successful. To date although clinical trials are underway, the benefit of targeting BRAF in pediatric LGG is as yet unproven.

## Author Contributions

CP and KK: manuscript research and writing. CF and SL: manuscript review and revision.

## Conflict of Interest Statement

The authors report no declarations of interest. The authors alone are responsible for the content and writing of the paper.

## Abbreviations

A, grade II astrocytoma; AA, anaplastic astrocytoma; CNS, central nervous system; DA, diffuse fibrillary astrocytoma; GBM, glioblastoma multiforme; GG, ganglioglioma; HGG, high-grade glioma; LGG, low-grade glioma; MAPK, mitogen-activated protein kinase; ODG, oligodendroglioma; PA, pilocytic astrocytoma; PFS, progression-free survival; PMA, pilomyxoid astrocytoma; PXA, pleomorphic xanthoastrocytoma; SGCA, subependymal giant cell astrocytoma.
